# Judging the Neonatal Abstinence Syndrome Assessment Tools to Guide Future Tool Development: The use of Clinimetrics as Opposed to Psychometrics

**DOI:** 10.3389/fped.2017.00204

**Published:** 2017-09-20

**Authors:** Philip M. Westgate, Enrique Gomez-Pomar

**Affiliations:** ^1^Department of Biostatistics, College of Public Health, University of Kentucky, Lexington, KY, United States; ^2^Division of Neonatology, Department of Pediatrics, University of Kentucky, Lexington, KY, United States

**Keywords:** Finnegan Neonatal Abstinence Score, formative model, Neonatal Abstinence Syndrome Score, predictive accuracy, reflective model

## Abstract

In the face of the current Neonatal Abstinence Syndrome (NAS) epidemic, there is considerable variability in the assessment and management of infants with NAS. In this manuscript, we particularly focus on NAS assessment, with special attention given to the popular Finnegan Neonatal Abstinence Score (FNAS). A major instigator of the problem of variable practices is that multiple modified versions of the FNAS exist and continue to be proposed, including shortened versions. Furthermore, the validity of such assessment tools has been questioned, and as a result, the need for better tools has been suggested. The ultimate purpose of this manuscript, therefore, is to increase researchers’ and clinicians’ understanding on how to judge the usefulness of NAS assessment tools in order to guide future tool development and to reduce variable practices. In short, we suggest that judgment of NAS assessment tools should be made on a clinimetrics viewpoint as opposed to psychometrically. We provide examples, address multiple issues that must be considered, and discuss future tool development. Furthermore, we urge researchers and clinicians to come together, utilizing their knowledge and experience, to assess the utility and practicality of existing assessment tools and to determine if one or more new or modified tools are needed with the goal of increased agreement on the assessment of NAS in practice.

## Introduction

The number of infants developing Neonatal Abstinence Syndrome (NAS) is reaching epidemic proportions with increasing number of affected infants receiving pharmacotherapy and increasing length of stay in hospital (1–4). Therefore, there has been much research recently addressing the assessment and management of infants with NAS. Unfortunately, there is considerable variability in practice with respect to both of these aspects (2, 5–8). Inherently, this implies an apparent lack of agreement among researchers and clinicians in terms of best methods.

In this manuscript, we focus on the assessment of infants with NAS. Multiple assessment tools have been developed and are in use, with the Neonatal Abstinence Syndrome Score, also known as the Finnegan Neonatal Abstinence Score (FNAS), being the most commonly used (5, 7, 9–12). However, the FNAS has been modified multiple times and is not used in exactly the same manner at every institution (5, 13). For instance, the MOTHER NAS score (MNS) (14, 15) made considerable revisions to the FNAS and may be increasing in popularity.

Due to the existence of multiple assessment tools that are implemented in practice, it is apparent that researchers and clinicians have not come to a common agreement on how to best assess NAS. Therefore, we address how to view the theoretical modeling framework for NAS from which assessment tools arise. Specifically, we contrast viewpoints based on psychometric (16–21) and clinimetric (17–19, 22) principles, concluding that NAS assessment tools should be viewed clinimetrically. We hope that conveying this modeling framework will give researchers and clinicians a realistic construct on how to properly judge NAS assessment tools, as utilizing a psychometric viewpoint will only lead to the conclusion that existing tools are invalid (21, 22). Ultimately, we hope that with this increased understanding on how to view NAS assessment, future work on creating or modifying assessment tools will lead to improved and standardized NAS assessment and thus management.

In the following section, we give relevant details on theoretical models corresponding to psychometric and clinimetric viewpoints, respectively, with regards to NAS assessment. We argue that a clinimetric-based formative model (17–19, 22) should be preferred, and then discuss how to judge tools and give examples from the literature. Finally, we provide a discussion on future tool development, followed by concluding remarks.

## Psychometric- and Clinimetric-Based Models for NAS Assessment

### Psychometrics—Reflective Model

**Figure 1:**
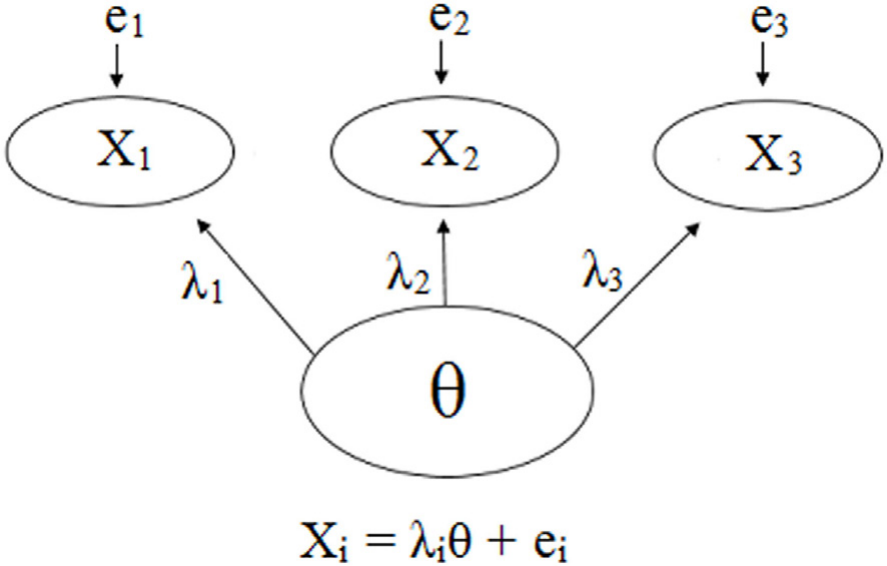
Visual and mathematical representations of a generic psychometrics-based reflective model in which items X_1_, X_2_, and X_3_ are caused by and hence are reflections of the hypothetical construct θ. Each item has its own equation in which the influence of θ is denoted by λ, and the items may have measurement error, *e*. The items are expected to be strongly correlated due to the influence θ has on each one. Note that the number of items is not restricted, and a scenario with three items was chosen for simplicity.

**Figure 2:**
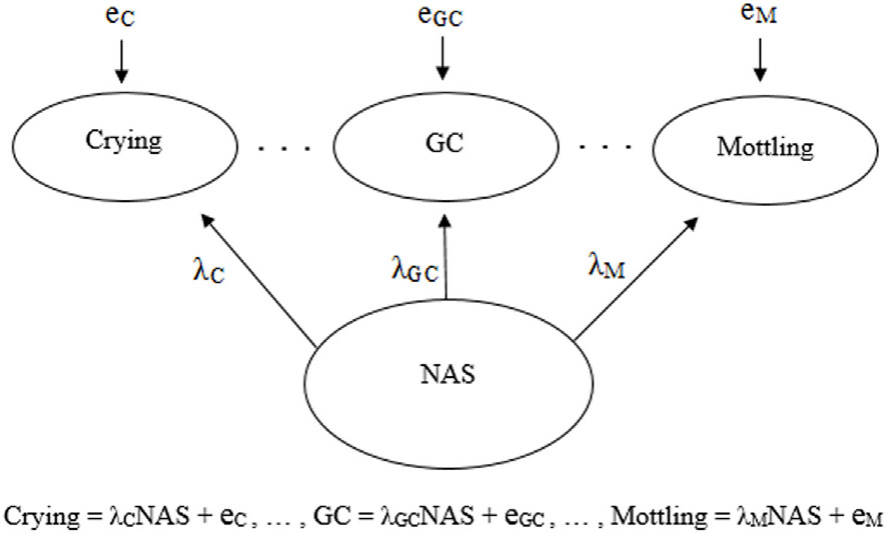
Visual and mathematical representations of a psychometrics-based reflective model in which crying, general convulsions (GC), mottling, etc. are caused by and hence are reflections of neonatal abstinence syndrome (NAS). Each symptom has its own equation in which the influence of NAS is denoted by λ, and the symptoms may have measurement error, *e*. The symptoms are expected to be strongly correlated due to the influence NAS has on each one.

Psychometric properties are regularly emphasized in practice (22), and hence, a reflective model (18, 19) is often used as the default to determine the validity of an assessment tool. Figures 1 and 2 demonstrate a generic reflective model and an assumed reflective model for NAS, respectively. Applying this model to NAS, it is assumed that NAS causes each item to present, and therefore, items should be a reflection of NAS and thus should be highly correlated; i.e., items should have good internal consistency or reliability (17–19). As a result, equal weighting of items is preferred (18, 23).

### Clinimetrics—Formative Model

**Figure 3:**
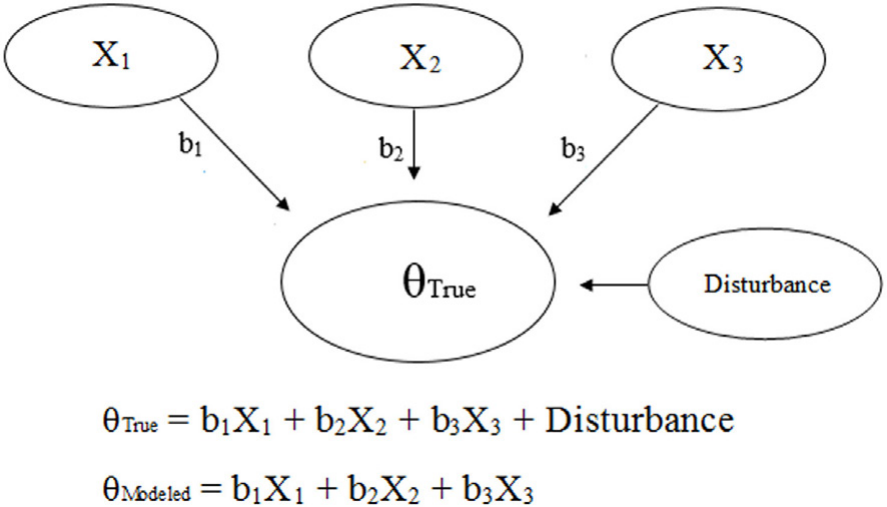
Visual and mathematical representations of a generic clinimetrics-based formative model in which items X_1_, X_2_, and X_3_ combine to form the true hypothetical construct θ_True_. The disturbance term represents the influence of all other factors besides X_1_, X_2_, and X_3_ on θ_True_. The numerical weights applied to the items in the assessment tool are represented by b_1_, b_2_, and b_3_. Note that the number of items is not restricted, and a scenario with three items was chosen for simplicity.

**Figure 4:**
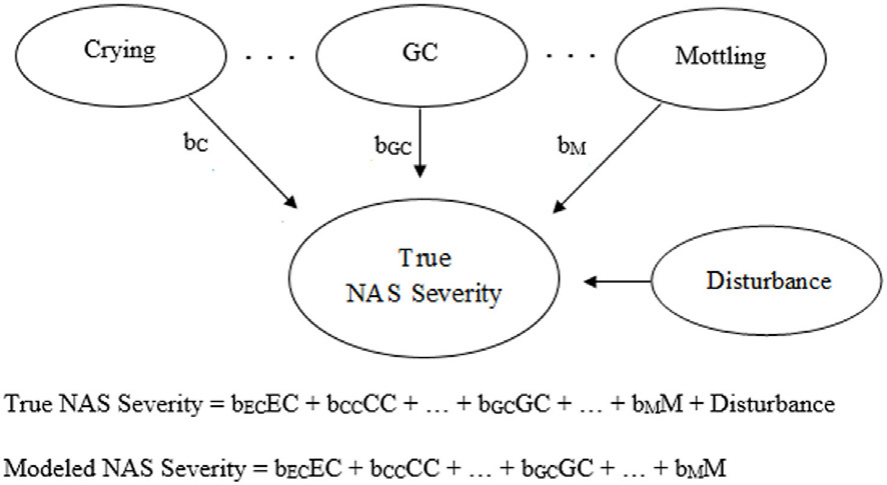
Visual and mathematical representations of a clinimetrics-based formative model in which crying (EC, excessive crying; CC, continuous crying), general convulsions (GC), mottling (M), etc. combine to form Neonatal Abstinence Syndrome (NAS) severity. The disturbance term represents the influence of all factors not accounted for by the given assessment tool. The numerical weights applied to the corresponding items in the assessment tool are represented by b_EC_, b_CC_, b_GC_, and b_M_. In the visual representation, b_C_ is equivalent to either b_EC_ or b_CC_, depending on the severity of crying.

Generic and NAS-based representations of formative models arising from clinimetrics-based reasoning are given in Figures 3 and 4, respectively. With this model, the items are not required to be correlated, and each item contributes, with potentially varying magnitudes, toward NAS severity (22). Specifically, the weights depicted in Figures 3 and 4 can vary in value according to item impact, and the weighted items are summed to form a modeled severity of NAS. Figure 4 specifically depicts the FNAS, which applies different numerical weighting for crying, general convulsions, mottling, etc. due to their perceived differing influences on NAS severity. In practice, the modeled severity may not be the true severity, implying a degree of discrepancy, also known as the disturbance, between the model and the truth. See, for instance (17–19), for specific details on formative models.

### Which Theoretical Modeling Framework Works Best for Judging NAS Assessment?

Table 1 provides a concise comparison of reflective and formative models. Evidence has shown that NAS assessment tools do not coincide with a reflective model. Admittedly, NAS can initially be thought of in terms of a reflective model, as NAS causes symptoms, and thus items in the FNAS, for instance, to present. However, the occurrence, severity, and duration of symptoms are quite variable across and within infants (13, 14, 21, 24), and thus do not reflect NAS to the same degree. As a result, Jones et al. (20) found *via* low Cronbach’s alphas on the FNAS and MOTHER NAS scale that not all items are highly correlated, and Bada et al. (25) also found *via* odds ratios that not all items are highly interrelated. We note that Cronbach’s alpha is a measure of the degree of correlation among a group of items in order to assess internal consistency (26).

**Table 1:** A comparison of reflective (psychometrics) and formative (clinimetrics) models.

Aspect	Reflective model	Formative model
Item role	All items are influenced by and, therefore, reflect Neonatal Abstinence Syndrome (NAS)	All items build up NAS severity
Item correlation	All items are highly correlated (internal reliability)	Items do not need to be correlated
Item weighting	Equal weighting	Unequal weighting, depending on perceived impact the item has on NAS severity

The signs of NAS, or symptoms if truly arising from NAS, build up NAS severity. Furthermore, clinical reality is that certain symptoms are more critical than others when determining the severity of NAS, and these symptoms and their severity may not highly correlate. Therefore, the formative modeling approach should be preferred as it provides a clinically meaningful basis on which NAS assessment tools can be judged. This approach also provides flexibility in that information other than symptom severity can be employed.

## Judging NAS Assessment *via* a Formative Modeling Framework

### General Approach

An ideal approach to obtaining a NAS assessment tool based on a formative approach would be to conduct an empirical study utilizing a large amount of clean or robust data from the target population, and to use that data to determine which items should be incorporated within the resulting tool and how much weight to assign each item. The primary interest in using tools to assess NAS severity is to determine when pharmacologic treatment is needed. Therefore, if we were able to obtain in this hypothetical study accurate data on when pharmacologic treatment is needed, logistic regression modeling could be employed to obtain estimated weights (see mathematical models presented in Figures 3 and 4) which indicate the importance of each item. The resulting tool could then be used on a separate large dataset of clean or robust data in order to judge and (hopefully) validate the predictive accuracy of the tool. An example of such an approach is the derivation of the Score for Neonatal Acute Physiology—Perinatal Extension (27).

Unfortunately, there is no way to determine true NAS severity and thus the need to treat, and therefore, we cannot judge tools based on their true predictive accuracy. As a result, assessment tools will need to be judged based on existing knowledge about NAS severity; i.e., we need to use knowledge from up-to-date research and clinical experience to make an educated guess as to what the ideal model for severity should look like. Essentially, judgments will need to be based on face validity (22), and we want a tool based on utility (e.g., good perceived predictive accuracy, or low disturbance based on Figures 3 and 4), practicality, and “clinical common sense” (22). Unfortunately, different people will have different judgments with respect to these considerations.

Specific judgments on a tool should be made with respect to three steps used to create an assessment tool based on a formative model (17). The first two steps consist of determining the potential variables to form the tool and ultimately deciding which variables, or items, should actually be utilized. The third step is to determine how much weight should be assigned to each item. Furthermore, judgments must be made on the value of pharmacologic treatment cutoff scores, and how to use them. For instance, the decision to treat when using the FNAS is often based on whether or not three scores, or their average, of 8 in a row are observed (9–11), thus also implying two 12s in a row. A summary of basic considerations, as well as differences in opinion that are seen in practice, is given in Table 2. Table 2 also summarizes issues in current practice as discussed in the remainder of this manuscript.

**Table 2:** Considerations and obstacles in the evaluation and creation of Neonatal Abstinence Syndrome (NAS) assessment tools.

***Basic considerations***
Visualize what the ideal model would look like using knowledge from up-to-date research and clinical experience – Consider face validity; i.e., perceived clinical utility – Consider practicality; e.g., scoring time – Consider pharmacologic treatment cutoff values based on the ideal model

***Differences in opinion***
People can have varying opinions on a perceived ideal model. For instance, opinions can vary with respect to: – the utility of an item; i.e., is the item needed in the tool, and if so, how much weight should be assigned to the item? – the practicality of the item; e.g., does the amount of time it takes to score the item outweigh its added utility to the tool? – what treatment cutoff value(s) should be used, and how should they be used? As a result of differences in opinion, perceived ideal assessment tools will also differ. This is one reason why new or modified assessment tools continue to be developed

***Issues in current practice***
Advances in research and differences in opinion continue to result in different or modified tools being proposed and used. Examples include: – additions and reductions in the utilized items, as in the MOTHER NAS score (14, 15) – shortened tools

Empirical research on treatment cutoffs has used NAS as the outcome of interest, as opposed to the true need to treat, and can, therefore, only provide suggestive evidence with respect to the need for treatment

Judgments should not only consider inter-rater reliability, but also the utility of items when deciding whether or not to include the item in a tool

Future tools should be developed based on a formative modeling strategy and can be created by adding or reducing items from existing tools. Furthermore, other information can be used in conjunction with such tools

Assessment tools based on formative modeling can be developed to encompass a variety of exposure types

Tools tend to only be developed by a small group of people before being published and presumably used. Due to likely differences in opinion from other clinicians and researchers, implementation and ultimately standardization in practice is unlikely

To help standardize NAS assessment, experts should come together to decide on the best formative model(s) and ultimately assessment tool(s) to use

## Examples

Although many people may not have realized the underlying theoretical framework, NAS assessment tools have been created *via* formative modeling approaches out of necessity. For instance, Finnegan et al. (9, 10) used the three steps as just discussed. In brief, common symptoms were chosen based on the literature and experience, and weights assigned to each item were chosen based on pathologic significance. Furthermore, the treatment cutoff was chosen based on experience.

The fact that multiple modified versions of the FNAS exist, and that not even treatment cutoff scores are used consistently across institutions (5, 6), demonstrates the fact that people have formatively judged the FNAS and have determined it could use change. Multiple reasons for this exist. As time goes on, our knowledge with respect to NAS improves, and therefore, the way true NAS severity is perceived may change, thus alternating the way we want to model the construct of NAS severity. For instance, the MNS was created by Jansson et al. (14) to improve upon the FNAS. Both irritability and failure to thrive, based on weight loss, were added whereas some items were removed or combined, for instance, due to overlap. Furthermore, the FNAS is lengthy and clinicians may desire a shorter tool (28) that is more practical and potentially more reliably scored, and therefore, shortened scores have been proposed (29). Even a short functional assessment approach has been developed which is based on feeding, sleeping, and ability to console when crying (30). We note that, unlike with a reflective model, heterogeneity of items in the assessment tool is clinically ideal as the use of correlated items unnecessarily adds time and complexity to scoring in practice.

An empirical approach that has been taken to formatively judge a NAS assessment tool, including its treatment cutoff value(s), is the use of data with the outcome of interest of whether or not the subject has NAS. Although this outcome is not ideal and will not provide definitive results because interest is actually in the unknown true need to treat, results can still be suggestive. For instance, Zimmerman-Baer et al. (31) studied 102 healthy neonates and found that the 95th percentile of their scores, from a version of the FNAS comprised of 28 items, never exceeded 8. Although this suggests that infants not in withdrawal will tend to have lower FNAS scores, and scores of 8 or above are mostly in infants who are withdrawing, this does not provide strong information with respect to the true need to treat infants in withdrawal. Specifically, interest is with respect to when NAS infants need to be treated, not if they actually have NAS. However, these particular results may be suggestive that, although a single score of 8 to deem treatment is needed is insufficient, an average of three 8s or two 12s in a row may suffice as adequate treatment cutoffs for this particular 28 item tool.

Although not a direct factor with respect to judging the formative nature of a tool, we feel it is important to note that the inter-rater reliability (IRR) of NAS assessment tools has received a notable amount of attention, as many items incorporated in NAS tools are based on subjective judgments. Although scorers should be trained, they may not always have complete agreement on the magnitude or occurrence of a clinical sign involved in a particular tool (23, 24). For instance, IRR coefficients have been shown, for example, to range from 0.70 (33) to 0.96 (9, 10) with the FNAS, 0.89 to 0.98 with the Neonatal Withdrawal Inventory (32), and greater than 0.94 with the MNS (5, 15). Furthermore, Gomez-Pomar et al. (33) showed that the degree of variance in FNAS scores attributable to unreliable scoring is small (≤9.8% in the studied institutions). Although when viewed from a psychometric viewpoint, an interobserver reliability of 90% is desired (21, 34), there may still be enough utility in keeping any items in the tool that cause lack in IRR as opposed to removing them. Therefore, judgments should not only consider IRR, but also the utility of items. For instance, although it may be difficult to clearly distinguish the cutoffs between mild, moderate, and severe tremors, incorporation of this item with the chance of misclassification can still be more useful than not using this item simply because of this potential difficulty.

## Future Tool Development

As more is learned about NAS, assessment tools may need to evolve. Based on a formative modeling approach, new items and their perceived impacts, *via* appropriate weights, can be added to tools, and numerical treatment cutoff values modified accordingly. Alternatively, items deemed to have little utility or practicality in the presence of other items can be removed. We do note that newer information, such as knowledge on genetics and the mother’s prenatal treatment (5), may be difficult to incorporate into a standard assessment tool. However, research could be conducted on how to best utilize such information in conjunction with the tool. For instance, Grossman et al. (30) recently demonstrated that a simple functional assessment can work well when combined with other novel strategies for NAS management.

Formative models can also conform to variations that exist in practice and that should be considered for future tool development. Presenting NAS symptoms may depend on the type of opioid causing withdrawal, among other possible factors (6, 13, 21, 35). Furthermore, many infants’ withdrawal can be a result from exposures to multiple types of drugs, which would not correspond to a single opioid-based tool. This issue is amplified when there is also exposure to non-opioids (21, 36). Extending upon the issue of heterogeneous exposures, variations in the distributions of exposure types may also exist across different institutions due to the different populations they care for (33). All of these variations will contribute to a lack in correlation among items in as assessment tool, and thus the tool will be deemed inappropriate if based on a reflective modeling viewpoint. However, when based on a formative model, a tool is allowed to encompass the spectrum of opioids. Specifically, this modeling approach allows us to simultaneously assess symptoms from different types of exposures, thus building up a case for the need to treat. This is important because it is clinically irrelevant to restrict the applicability of a tool to a single exposure type when in real life we are faced with multidrug-exposed withdrawing infants (37). We do note, however, that this issue adds a notable degree of complexity in terms of judging the utility of any such tool, and multiple tools may be required; e.g., a unique tool for a single or specific mixture of exposures, or even a unique tool for institutions observing similar distributions in exposures. Unfortunately, no existing or future tools will be ideal, but we hope to obtain the best guidance as practically possible from these tools (28).

Major contributing factors to the existence of a variety of NAS assessment tools include the fact that researchers have been independently judging and creating these tools and differences in opinion exist. As a result, it is very difficult for any new tool to be widely assessed and ultimately implemented in practice, as other clinicians and researchers may not agree with the proposed tool in terms of its utility and practicality. Therefore, we urge that a large group of experts in the area of NAS assessment should come together and pool their knowledge and clinical experience to assess the utility and practicality of existing assessment tools and to determine if one or more new or modified tools are needed. If needed, such “best” tools should be formulated with consideration of both perceived utility and practicality based on a formative modeling strategy. We suspect there will never be complete agreement and, therefore, compromises and ultimately a consensus in terms of the included items and the assigned weights to each item is likely needed (17). Furthermore, randomized clinical trials may be warranted to confirm the superiority of any such tools to existing tools on outcomes of importance such as length of stay and duration of opioid treatment (13, 38).

## Conclusion

There is push in the NAS literature on the need for a new assessment tool that is psychometrically valid (21). However, this inherent proposed use of a reflective model does not correspond to the real-world diversity of NAS manifestations observed in practice and the fact that numerical scores from an assessment tool do not completely dictate the decision to start pharmacological treatment. Therefore, in this manuscript, we argue that judgments on, and therefore future development of, NAS assessment tools should be based on a formative modeling approach. Such an approach can take into account the complexity of the presenting symptoms of NAS, their severity, and potentially the fact that heterogeneity of symptoms and their severity exists across different types and mixtures of opioid exposures. Finally, current assessment tools must be judged using current knowledge and experience, and judgment can vary in opinion. Therefore, one or more assessment tools based on a formative model and created based on the input of a large group of experts may be needed in order to lead to increased agreement on the assessment of NAS in practice.

## Author Contributions

PW conceived the work, drafted and revised the manuscript, approved the final manuscript as submitted, and agreed to be accountable for all aspects of the work. EG-P assisted in the conception of the work, made critical input and assisted in revisions, approved the final manuscript as submitted, and agreed to be accountable for all aspects of the work.

## Conflict of Interest Statement

The authors declare that the research was conducted in the absence of any commercial or financial relationships that could be construed as a potential conflict of interest.
